# Dihydroxyacetone of wheat root exudates serves as an attractant for *Heterodera avenae*

**DOI:** 10.1371/journal.pone.0236317

**Published:** 2020-07-23

**Authors:** Gaofeng Wang, Yunhe Wang, Hazem Abdelnabby, Xueqiong Xiao, Wenkun Huang, Deliang Peng, Yannong Xiao

**Affiliations:** 1 Key Laboratory of Plant Pathology of Hubei Province, College of Plant Science & Technology, Huazhong Agricultural University, Wuhan, Hubei, China; 2 State Key Laboratory for Biology of Plant Diseases and Insect Pests, Institute of Plant Protection, Chinese Academy of Agricultural Science, Beijing, China; Consiglio per la Ricerca e la Sperimentazione in Agricoltura, ITALY

## Abstract

*Heterodera avenae*, as an obligate endoparasite, causes severe yield loss in wheat (*Triticum aestivum*). Investigation on the mechanisms how *H*. *avenae* perceives wheat roots is limited. Here, the attractiveness of root exudates from eight plant genotypes to *H*. *avenae* were evaluated on agar plates. Results showed that the attraction of *H*. *avenae* to the root exudates from the non-host *Brachypodium distachyon* variety Bd21-3 was the highest, approximately 50 infective second-stage juveniles (J2s) per plate, followed by that from three *H*. *avenae*-susceptible wheat varieties, Zhengmai9023, Yanmai84 and Xiangmai25, as well as the resistant one of Xinyuan958, whereas the lowest attractive activity was observed in the two *H*. *avenae*-resistant wheat varieties, Xianmai20 (approximately 12 J2s/plate) and Liangxing66 (approximately 11 J2s/plate). Then Bd21-3, Zhengmai9023 and Heng4399 were selected for further assays as their different attractiveness and resistance to *H*. *avenae*, and attractants for *H*. *avenae* in their root exudates were characterized to be heat-labile and low-molecular compounds (LM) by behavioral bioassay. Based on these properties of the attractants, a principle of identifying attractants for *H*. *avenae* was set up. Then LM of six root exudates from the three plants with and without heating were separated and analyzed by HPLC-MS. Finally, dihydroxyacetone (DHA), methylprednisolone succinate, embelin and diethylpropionin in the root exudates were identified to be putative attractants for *H*. *avenae* according to the principle, and the attraction of DHA to *H*. *avenae* was validated by behavioral bioassay on agar. Our study enhances the recognition to the orientation mechanism of *H*. *avenae* towards wheat roots.

## Introduction

Cereal cyst nematode, *Heterodera avenae* is one of the most important nematode pests that limits production of small grain cereals. Heavily invaded young plants are stunted and leaves are often chlorotic, forming pale green patches in the field [1]. It is believed that attractive cues of plant root exudates for nematodes mediate the recognition between plants and nematodes, and the nematode sense organs, amphids functioning as chemoreceptors are mainly responsible for detecting the chemochemicals [2–4]. In addition to this function, root exudates also play crucial roles in other biological processes. For instance, root exudates can increase nematode egg hatching rate [5, 6], and plant can alter its rhizosphere microbial community structure and diversity through its root exudates [7, 8].

Biotic and abiotic factors have effects on the components of plant root exudates. In tall fescue, its endophytical fungus, *Neotyphodium coenophialum* synthesizes special alkaloids in roots altering the chemotaxis of the pathogenic nematode, *Pratylenchus scribneri* to the roots [9]. The arbuscular mycorrhizal fungus, *Funneliformis mosseae*, can change tomato root exudates, and this results in the reduction of the penetration of *Meloidogyne incognita* on tomato roots [10]. Similarly, tomato root exudates could also be modified by *M*. *incognita*, leading to contents of the four compounds, 2,6-Di-tert-butyl-p-cresol, L-ascorbyl 2,6-dipalmitate, dibutyl phthalate and dimethyl phthalate, in the root exudates being increased [11]. Meanwhile, the abiotic factor exogenous thiamine that was drenched on rice roots could weaken *M*. *graminicola* attraction to the roots [12], indicating the modification of rice root exudates caused by thiamine. Abiotic factors may alter root exudates through modulating phytohormone signaling, as phytohormones were found to control nematode responses to plant roots [13, 14].

Some attractants of plant root exudates for plant-parasitic nematodes (PPNs) have been identified, including CO_2_, g-aminobutyric acid, ions, glucose and so on [4, 15, 16]. Among these attractants, CO_2_ can attract free-living, entomopathogenic and plant parasitical nematodes [4], and CO_2_ seems to be a common attractive cue of plants for PPNs. It was found that *M*. *hapla* is attracted to pH gradients between pH 4.5 and 5.4, which implies the attractiveness of CO_2_ for nematodes may be due to acidification of solutions by dissolved CO_2_ [17]. This may also be the underlying mechanisms of some organic acids in root exudates attracting PPNs. In addition to attractants, there are also repellents of plant root exudates for PPNs, and attractiveness of root exudates to PPNs are determined by the combined effect of attractants and repellents, where the chemotactic responses of PPNs to roots depend on the ratios of attractants to repellents in the root leachates [18–20]. Moreover, the responses of PPNs to their attractants are concentration-dependent [16, 21].

Although researches on chemotaxis of PPNs to host roots have made significant progress, it remains elusive how PPNs recognize plant roots. The main objective of our works was to identify attractive cues of wheat (*Triticum aestivum*) root exudates for *H*. *avenae* through behavioral bioassay and HPLC-MS assay, which increases the recognition to the orientation mechanism of *H*. *avenae* towards wheat roots.

## Materials and methods

### Nematode culture and isolation

Cereal cyst nematode *Heterodera avenae* was maintained on a compatible wheat (*Triticum aestivum*) variety Zhengmai9023 at 16 °C in a greenhouse. Cysts were harvested from in *vitro* stock cultures and eggs in the cysts were hatched at 16 °C after at least a month incubation at 4 °C. Then freshly hatched second-stage juveniles (J2s) outside the cysts were collected.

### Preparations of root exudates

Eight plant genotypes were used in this study, including a non-host *Brachypodium distachyon* variety (Bd21-3), four resistant (Xinmai20, Xinyuan958, Heng4399, Liangxing66) and three susceptible (Zhengmai9023, Xiangmai25 and Yanmai84) wheat varieties toward *H*. *avenae*. Root exudate of each variety was collected from 300 seedlings by a standard preparation that was used repeatedly throughout these studies. Seeds were sterilized in 0.5% NaClO solution for 5 min, washed thoroughly with distilled sterile water for three times and transferred to fifteen-cm-diameter petri plates containing distilled sterile water and filter papers. After 3 days of incubation in the dark at 25 °C, seedlings (about 3 cm in height) were transferred to three boxes with 300 mL 1/2 MS solution for further growth under the condition of 16 h light/8 h dark at 25 °C. After 30 days, seedling roots were washed with distilled sterile water for four times, and transferred to new boxes with distilled sterile water (200 mL) for further growth under the same conditions. After 3 days, the root exudates solution was passed through a 0.45-μm filter, followed by freeze-drying for 3 days at -50 °C. Finally, the root exudate of each variety was dissolved with distilled sterile water (1 mg/mL) and stored at 4 °C.

For characterizing the physical and chemical properties of attractants for *H*. *avenae* in root exudates of Bd21-3, Heng4399 and Zhengmai9023 prepared above, each root exudate underwent two different treatments, respectively. In the first treatment, each root exudate (100 μL) was heated at 50 °C, 75 °C and 100 °C for 15 min in a tube sealed, respectively, using correspondingly original root exudate solutions (ORE) and distilled sterile water as positive and negative controls. In the second treatment, the low- and high-molecular weight compounds (LM and HM) of each root exudate (100 μL) were isolated with Zeba^™^ Spin Desalting Columns (Thermo, Cat. No. 89882) according to its protocol, and dissolved with distilled sterile water (100 μL), using corresponding ORE and distilled sterile water as positive and negative control. For HPLC-MS assay, LM were isolated from root exudate solutions prepared (200 μL) as described above, and dissolved with distilled sterile water (200 μL). Then LM (100 μL) was heated at 100°C, using the LM without heating (100 μL) as reference. Finally, all the root exudates were freeze-dried for 3 days and dissolved in methanol (100 μL). All the treated root exudate solutions were stored at 4 °C for use.

### Chemotaxis assay

The responses of *H*. *avenae* to ORE and that with different treatments, as well as dihydroxyacetone (DHA; Sigma, Cat. No. PHR1430) with different concentrations (0.1 mg/L, 0.5 mg/L and 1.0 mg/L) were analyzed. Five-cm-diameter Petri dishes containing 5 mL of 0.5% agar were used, where two circular marks (1 cm diameter) were made on each plate bottom that were both 1.5 cm away from the plate center. The root exudates and DHA (10 μL) that were screened was added into 1.5 mL tube containing 0.5% agar (200 μL), respectively. After the agar becoming to be solid, it was placed on the agar surface over the center of one circle, and 0.5% agar without samples over the second one was set up as negative control. *H*. *avenae* J2s (100 J2s/plate) were placed in the center of the plate. The numbers of J2s around agar containing the sample tested within 2 mm were counted at 12 h after nematodes being placed. There were five replicates for each experiment and two biological repeats.

### HPLC-MS assay

**Fig 1:**
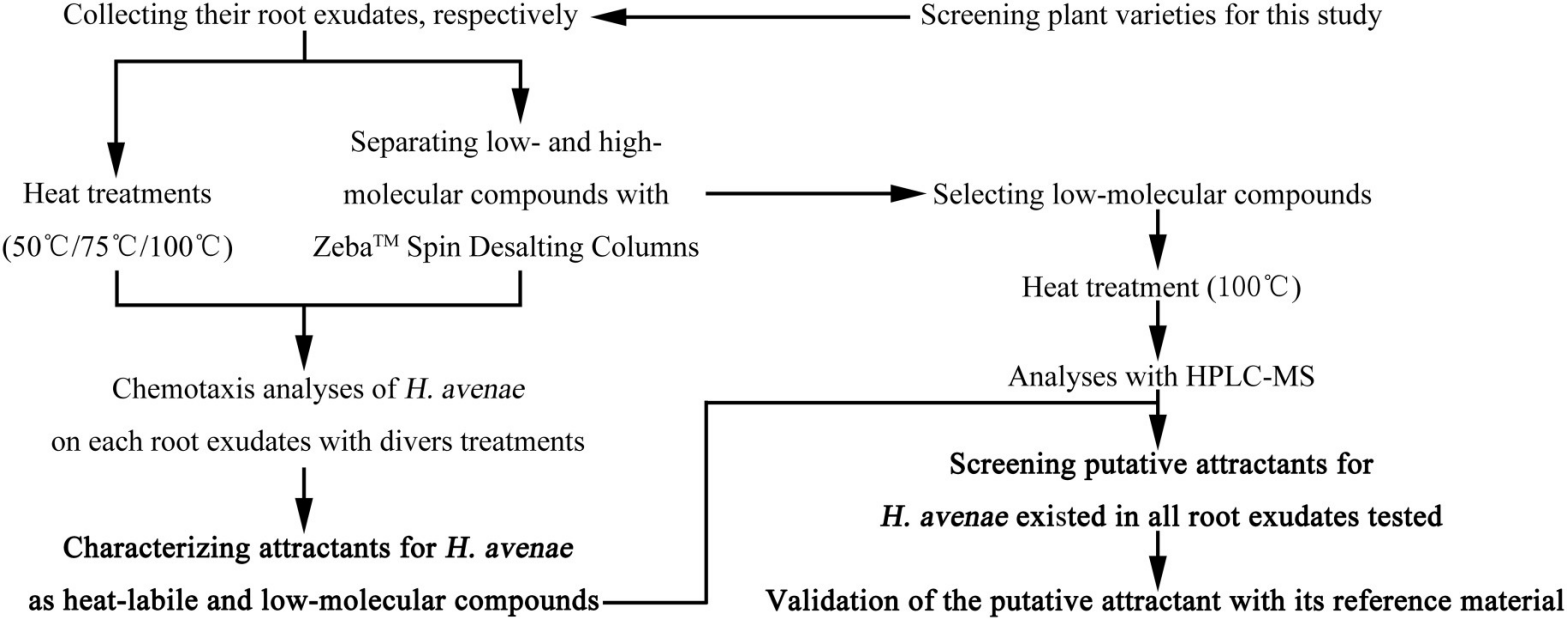
Schematic view of identifying attractants of root exudates for *H*. *avenae*.

The principle of identifying the attractants from root exudates tested for *H*. *avenae* was set up, where root exudates of a non-host of *H*. *avenae*, *B*. *distachyon* (Bd21-3), four *H*. *avenae*-resistant and three *H*. *avenae*-susceptible wheat varieties were collected, and attractants for *H*. *avenae* in these root exudates were identified that were presumed to exist in all the eight root exudates (Fig 1). Details on this principle are as follows. Firstly, the attractiveness of root exudates from the eight plants to *H*. *avenae* was assessed, respectively, and three of them with different resistance levels and high attractiveness to *H*. *avenae* were selected for the next analyses. Secondly, the physical properties of putative attractants for *H*. *avenae* in the three root exudates were characterized, including their molecular weights and thermal stabilities. For analyzing their molecular weights, low- and high-molecular compounds (LM & HM) in the three root exudates were isolated with Zeba^™^ Spin Desalting Columns, respectively. Meanwhile, the three root exudates were heated at 50°C, 75°C and 100°C, respectively to prepare different root exudates used for analyzing thermal stabilities of the putative attractants. Then, chemotactic responses of *H*. *avenae* to all the root exudates prepared above were assayed, using the corresponding OREs as controls. Finally, putative attractants for *H*. *avenae* in root exudates of the three varieties were determined to be LM and heat-labile by comparing the attractive activities to *H*. *avenae* among ORE, LM and HM and among root exudates with and without heating of each variety prepared above, respectively. Thirdly, LMs of the three varieties were heated at 100°C, and identifications and quantifications of these LMs with and without heating were performed through HPLC-MS assay. Finally, putative attractants for *H*. *avenae* in root exudates of the three varieties were identified that existed in all the three root exudates, and contents of which were decreased after heating. Putative attractants were validated with their reference materials.

The HPLC-MS assay of each root exudate sample was performed as follows. Samples (1.0 μL/sample) were subjected to HPLC-MS assay. Using an XDB-C18 column, HPLC analysis was performed with acetonitrile and 2.0% (v/v) acetic acid (pH = 2.59) as mobile phase at a flow rate of 1.0 mL/min. The temperature conditions were as follows: Ion source: 230 °C; inlet: 250 °C. Ionization mode: EI, electron energy 70 eV; mass scan range: 29–350 m/z, solvent delay time 4.5 min. The various components were identified from the retention times and mass spectra using the reference material from Mass Spectral Library. Quantification was performed using an internal standard, ribitol (99%, Sigma-Aldrich Chemie, Steinheim, Germany) that was added to each sample.

### Statistical analysis

All data were expressed as mean ± SE. The experimental data were analyzed by Tukey’s test that was conducted with IBM SPSS software (Version 30.0, SPSS Inc., Chicago, IL, USA). Values with *P* < 0.01 were considered statistically significant.

## Results

### Attractions of *H*. *avenae* to root exudates from eight different plant varieties were characterized

**Fig 2:**
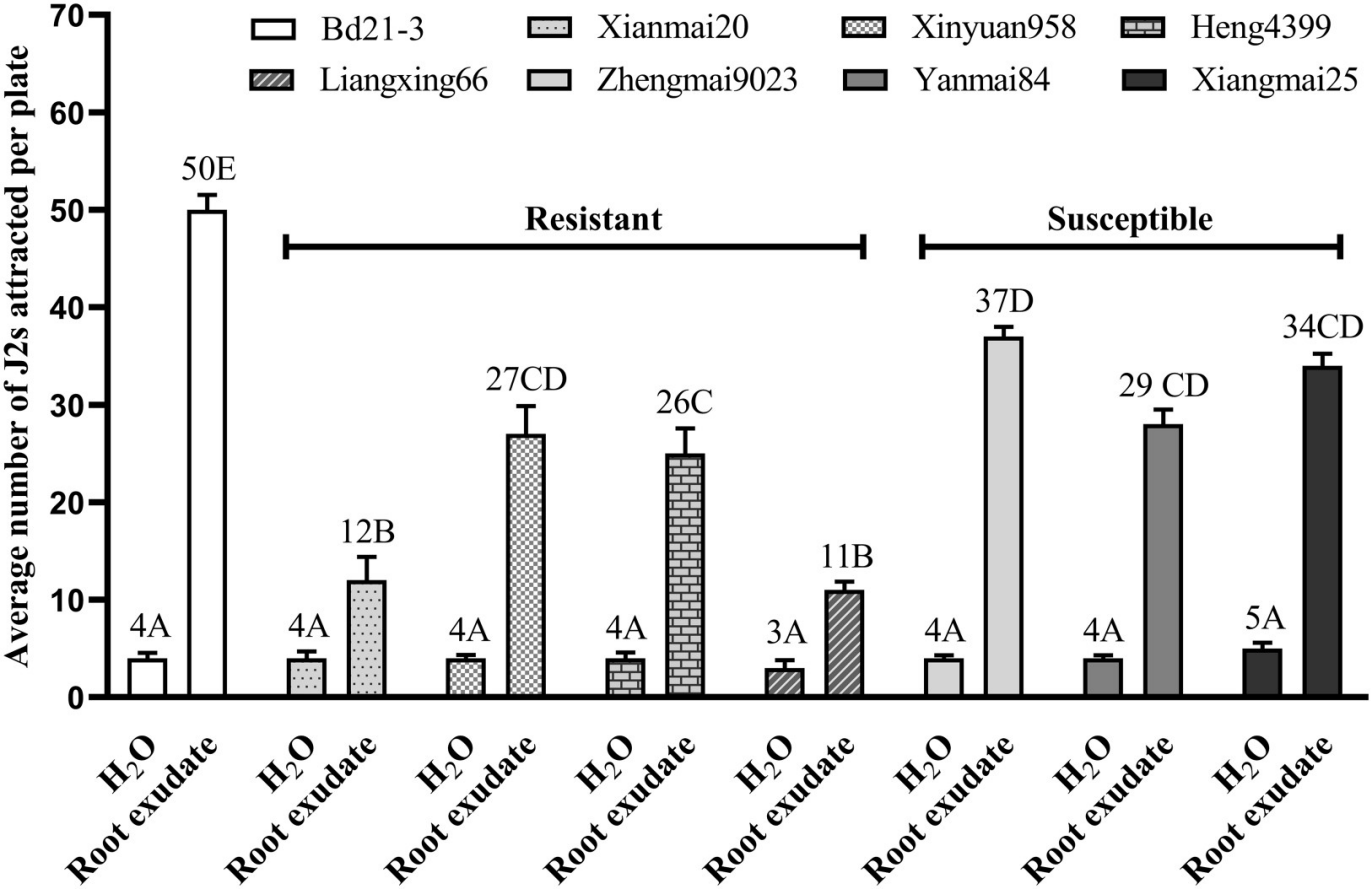
Attraction levels of *H*. *avenae* to different root exudates. Root exudates from a non-host of *H*. *avenae B*. *distachyon* variety (Bd21-3), six wheat varieties (Xinmai20, Xinyuan958, Heng4399, Liangxing66, Xiangmai25 and Zhengmai9023) and an Oat variety (Yanmai84) were collected. Attraction of *H*. *avenae* to each root exudate was tested, respectively, using distilled sterile water as negative control. Different letters above the bars denote a significant difference (*P* < 0.01). Values were means ± SE, n = 5.

Out of all the eight root exudates from different plants, that from *B*. *distachyon* variety Bd21-3 showed the highest attractive activity to *H*. *avenae*, which followed by the three susceptible wheat varieties Zhengmai9023, Yanmai84 and Xiangmai25 and a resistant wheat variety Xinyuan958 (*P* < 0.01; Fig 2). The lowest attraction level of *H*. *avenae* to root exudates were observed in the two *H*. *avena*e resistant wheat varieties, Xianmai20 and Liangxing66 (*P* < 0.01; Fig 2).

### Attractants of root exudates tested for *H*. *avenae* were heat-labile and low molecular compounds

**Fig 3:**
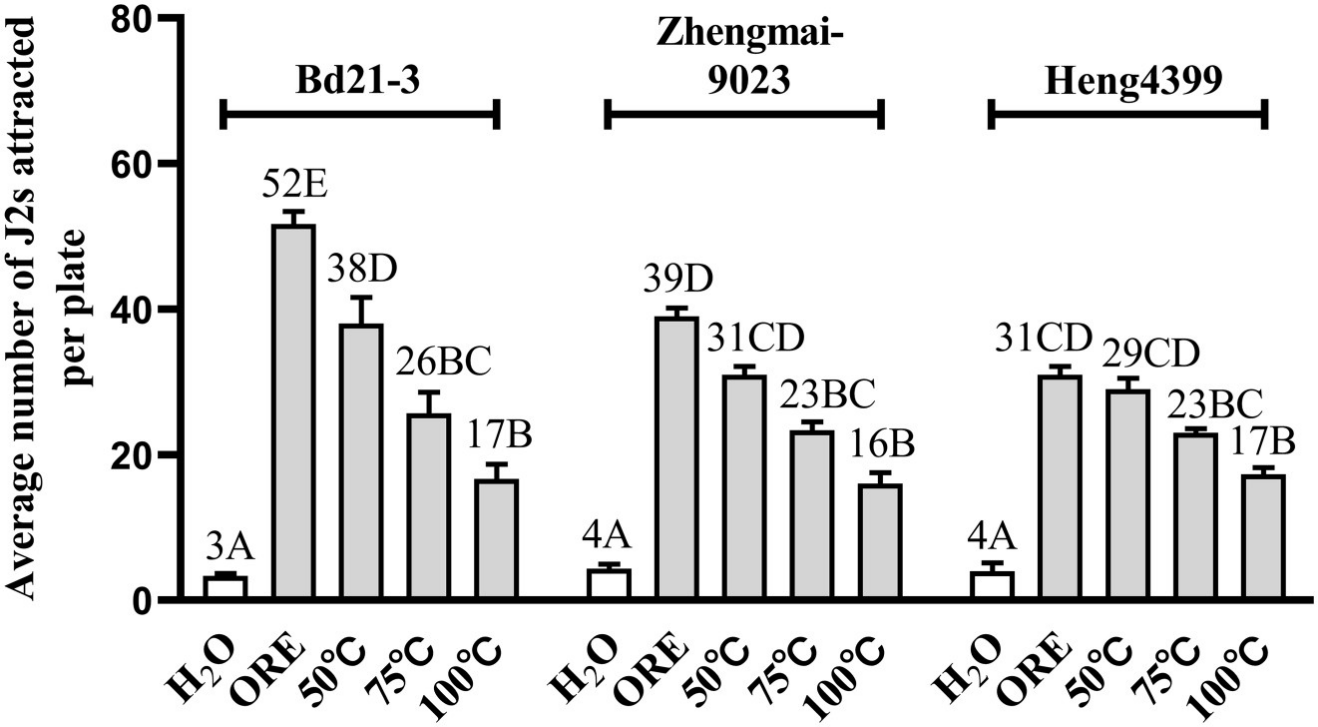
Chemotactic responses of *H*. *avenae* to root exudates with different heat-treatments. Root exudates of the *B*. *distachyon* variety Bd21-3 (non-host) and two wheat varieties Zhengmai9023 (susceptible) and Heng4399 (resistant) were heated at 50 °C, 75 °C and 100 °C for 15 min, respectively. Then the attractiveness of each root exudate for *H*. *avenae* was tested, respectively, using original root exudates (ORE) and distilled sterile water as positive and negative controls. Different letters above the bars denote a significant difference (*P* < 0.01). Values were means ± SE, n = 5.

Physical and chemical properties of putative attractants for *H*. *avenae* in root exudates from Bd21-3, Zhengmai9023 and Heng4399 were characterized. Results showed that attraction levels of *H*. *avenae* to the root exudates that were heated at 50 °C were significantly reduced in that of Bd21-3, but not for Zhengmai9023 and Heng4399 (*P* < 0.01; Fig 3). Meanwhile, root exudates heated at 100 °C resulted in lower attractive activities to *H*. *avenae* for all the three plant root exudates (*P* < 0.01; Fig 3). These data elucidated that the putative attractants that were present in all the three plant root exudates for *H*. *avenae* were heat-labile.

**Fig 4:**
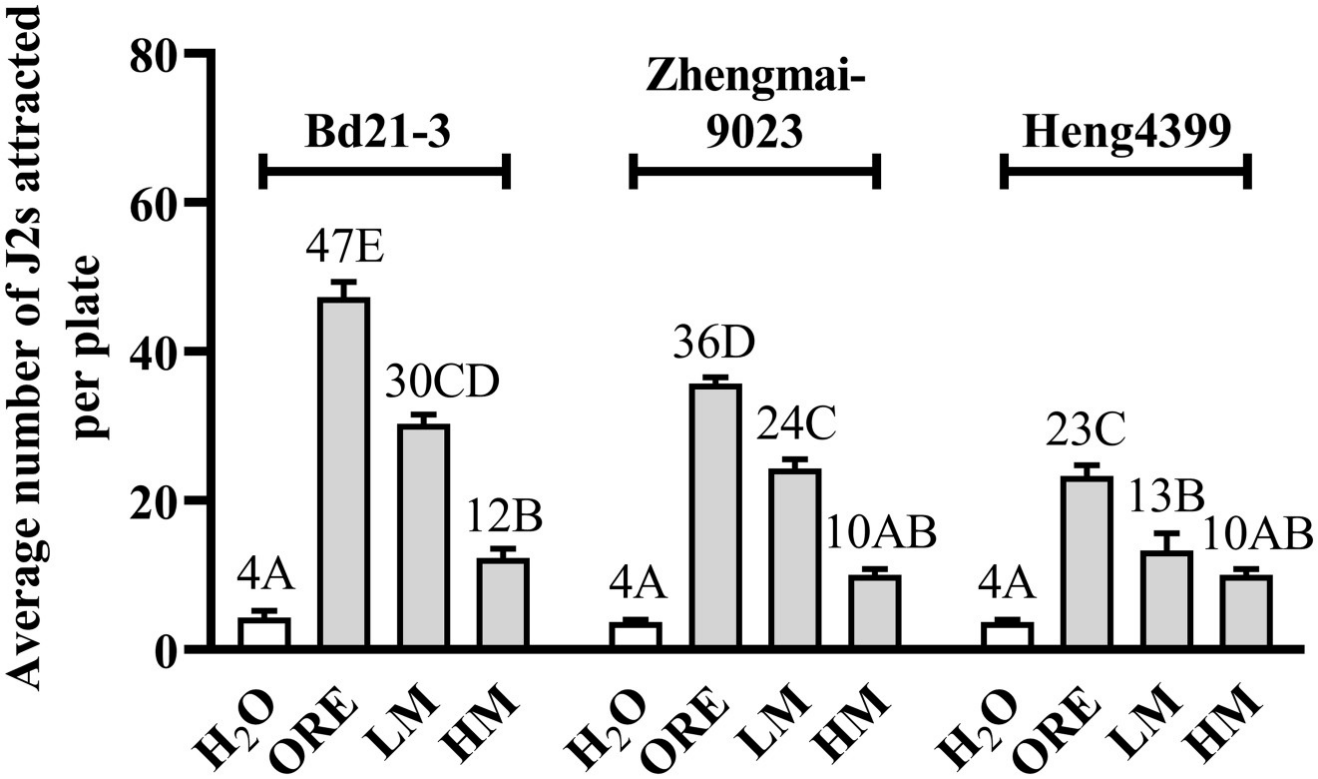
Chemotactic responses of *H*. *avenae* to compounds with different molecular weights isolated from the three root exudates tested. Compounds with low- and high- molecular weight (LM and HM) were isolated from the root exudates of the *B*. *distachyon* variety Bd21-3 (non-host) and two wheat varieties Zhengmai9023 (susceptible) and Heng4399 (resistant) with Zeba^™^ Spin Desalting Columns, respectively. Then the attractiveness of these original root exudate solutions (ORE), LM and HM for *H*. *avenae* were tested, using distilled sterile water as negative control. Different letters above the bars denote a significant difference (*P* < 0.01). Values were means ± SE, n = 5.

In addition, low-molecular compounds (LM) showed higher attractive activities to *H*. *avenae* than high-molecular compounds (HM) for the two root exudates from Bd21-3 and Zhengmai9023, but not for that from Heng4399, where the attractiveness of LM to *H*. *avenae* was approximately 30 J2s/plate, 24 J2s/plate and 13 J2s/plate, respectively (*P* < 0.01; Fig 4). It was implied that attractants included in all the three root exudates for *H*. *avenae* were also low-molecular compounds. However, the highest attractive activities to *H*. *avenae* were observed at original root exudates for all the three plants (*P* < 0.01; Fig 4).

### Four putative attractants for *H*. *avenae* were identified in root exudates

Based on the principle of identifying the attractants from root exudates tested for *H*. *avenae*, the *B*. *distachyon* variety Bd21-3 and two wheat varieties (Heng4399 and Zhengmai9023) were selected to screen the putative attractants for *H*. *avenae* for their different resistance levels and high attractions of *H*. *avenae* to their root exudates described above. In addition, for the putative attractants were low-molecular and heat-labile compounds, the putative attractants were identified by screening low-molecular compounds that existed in all the three root exudates of Bd21-3, Heng4399 and Zhengmai9023, of which the contents were declined after being heated at 100 °C as they are heat-labile. Results of HPLC-MS assay showed that 60, 25 and 29 different compounds were identified in root exudates of Bd21-3, Heng4399 and Zhengmai9023, respectively, including organic acid, aldehyde, amino acid and so on, and 32, 22 and 11 different compounds were reduced after heated, respectively (S1-S3 Tables). Finally, four putative attractants for *H*. *avenae* were identified, which were Dihydroxyacetone (DHA), methylprednisolone succinate, embelin and diethylpropion (Table 1).

**Table 1:** Putative compounds mediating the chemotaxis of *H*. *avenae* on three root exudates tested.

Compound	Molecular formula	Reduction of quantitate (%)^a^		
		Bd21-3	Zhengmai9023	Heng4399
Dihydroxyacetone	C_3_H_6_O_3_	30.48	100.00	100.00
Methylprednisolone succinate	C_26_H_34_O_8_	15.49	93.14	33.33
Embelin	C_17_H_26_O_4_	9.09	41.79	26.00
Diethylpropion	C_13_H_19_ NO	12.00	45.95	21.95

^a^The reduction of quantitate of each compound after being heated at 100 °C were calculated using their correspondingly low-molecular weight compounds without heating as references.

### DHA validation as an attractant for *H*. *avenae*

**Fig 5:**
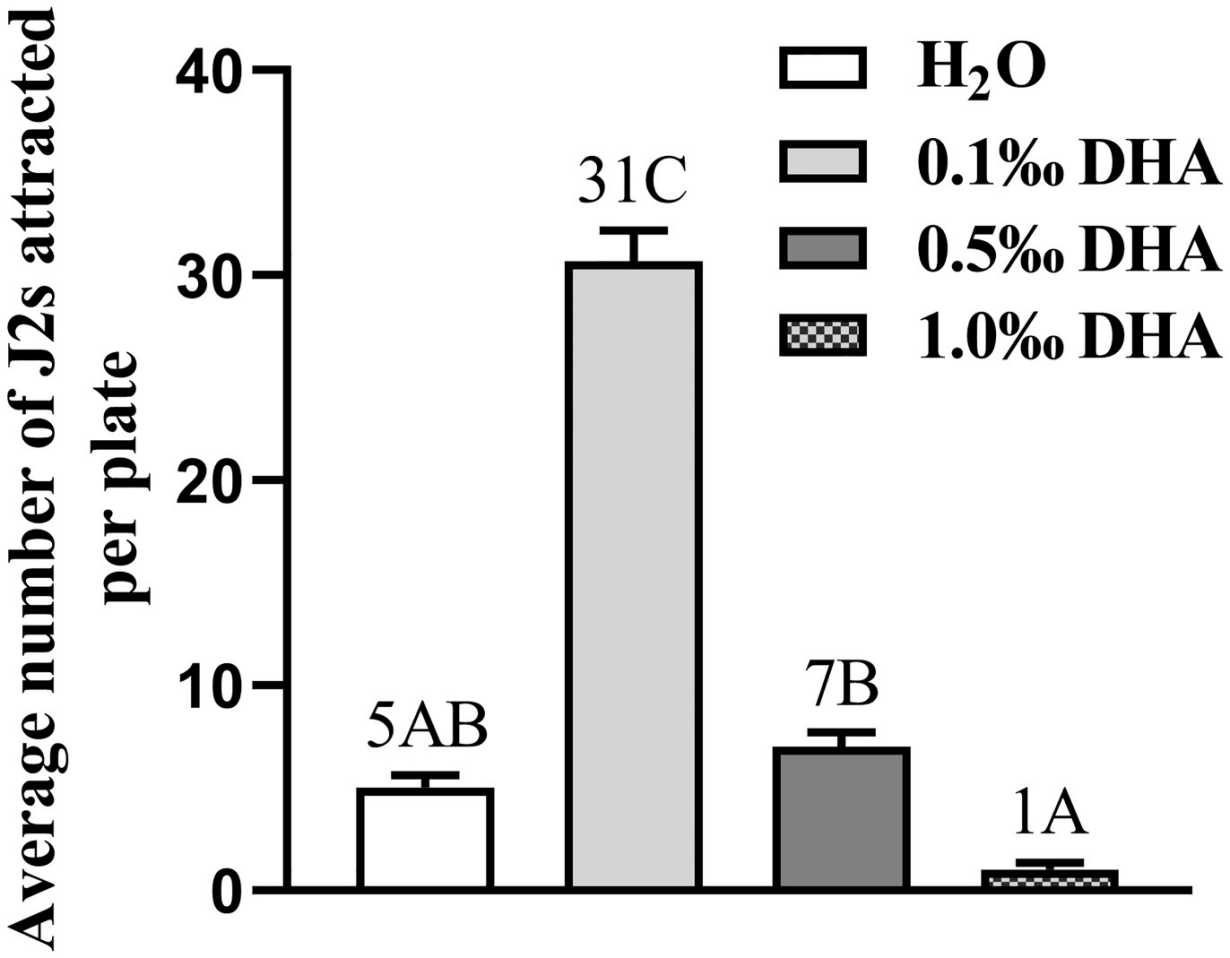
Chemotaxis of *H*. *avenae* to DHA with different concentrations. Three DHA solutions with different concentrations were prepared with DHA reference material. Then the attractive activities of the three DHA solutions to *H*. *avenae* were tested, using distilled sterile water as negative control. Different letters above the bars denote a significant difference (*P* < 0.01). Values were means ± SE, n = 5.

Using the reference material of DHA, chemotaxis of *H*. *avenae* to DHA was validated by behavioral bioassay on agar. Compared with sterilized distilled water, significant attractive activity of DHA to *H*. *avenae* was observed (approximately 31 J2s/plate) at the concentration of DHA being 0.1 mg/L, but not for that being 0.5 mg/L and 1.0 mg/L (Fig 5). In addition, higher attractiveness of 0.5 mg/L DHA for *H*. *avenae* was observed compared with that of 1.0 mg/L DHA. These data implied that the attractiveness of DHA for *H*. *avenae* was concentration-dependent.

## Discussion

It is well documented that host root exudates play important roles in the orientation of host roots for PPNs, whereas recognitions to the mechanisms are limited [22]. In this study, it was found that there was no detectable correlation between attractiveness and resistance of the plants to *H*. *avenae*, as the highest attractive activity of root exudates to *H*. *avenae* was observed in the non-host of *H*. *avenae*, *B*. *distachyon* variety Bd21-3, which followed by the four wheat varieties with and without resistance to *H*. *avena*e. The similar result is observed in banana, where both the susceptible and resistant banana varieties towards *Radopholus similis* attract this nematode [23]. It was also deduced that certain common attractive cues for *H*. *avenae* existed in all root exudates tested in this study. The postulation is supported by the findings of CO_2_ serving as attractant for *Panagrellus silusiae*, *Ditylenchus dipsaci*, *M*. *incognita* and other below-ground living herbivores [24–26]. It is a logical hypothesis that the common attractants for *H*. *avenae* in both *B*. *distachyon* and wheat root exudates are most likely to be found in their root exudates with higher attractiveness to *H*. *avenae*, although the chemotaxis of PPNs to root exudates are determined by both attractants and repellents for PPNs in root exudates [20, 22, 27]. According to the speculation, the *B*. *distachyon* variety Bd21-3 and two wheat varieties Zhengmai9023 and Heng4399 were selected for further identifications of attractive cues for *H*. *avenae* because of their higher attractive activities and different resistance to *H*. *avenae*.

In addition, the attractants for *H*. *avenae* in root exudates from the three plants were characterized to be heat-labile and LM. Attractants for nematodes with the heat-labile character are also found in soybean and citrus root exudates [28–30]. However, attractants for PPNs in root exudates with different physical and chemical properties from that found in this study are described, such as that in onion exudate roots [31]. Based on the properties of the target attractants, LMs in root exudates of Bd21-3, Zhengmai9305 and Heng4399 were further identified and quantified by HPLC-MS. There are limited component assays for *B*. *distachyon* root exudates [32]. Several works refer to the GC-MS assays of wheat root exudates [33–35], where the materials found in the root exudates are different from that we found in this study. It might be due to the different assay methods. Our data provides intriguing insights into the recognitions to the components of *B*. *distachyon* and wheat root exudates.

Finally, four putative attractants were identified, including DHA. However, none of them has been reported as attractive cue of plant to pests, including PPNs. The three putative attractants, methylprednisolone succinate, embelin and diethylpropion, are applied in pharmacological studies and medical treatments [36–38], whereas mass productions of these three compounds are unavaible. However, DHA is widely used in food processing industry, cosmetic industry and medical field as a kind of important chemical product [39], indicating its mature mass production, which makes DHA show greater application potential in *H*. *avenae* control compared with the three other putative attractants. The attractiveness of DHA to *H*. *avenae* was assessed at concentrations ranged from 0.1 μg/mL (20 ng/plate) to 1.0 μg/mL (200 ng/plate). It has been reported that contents of wheat root exudate compounds analyzed ranged from 1.35 ng to 54.15 ng per root system [40]. It’s a reasonable deduction that concentrations of DHA used in this study reflect the concentration of DHA in wheat root exudates. It was found that like other attractants for nematodes reported [16, 41], the response of *H*. *avenae* to DHA was concentration-dependent, because attraction levels of *H*. *avenae* to DHA with concentrations of 0.1 mg/L, 0.5 mg/L and 1.0 mg/L were different. Whether DHA plays roles in the orientations of plant for other nematodes remains unknown. In this study, the attractiveness of DHA and the full exudate solutions to *H*. *avenae* was not compared. It was speculated that higher attractive activity will be observed in the full exudate solutions. Taking together, our study increases the recognition to the orientation mechanism of *H*. *avenae* towards wheat roots, indicating the potential application of DHA, as an attractant to *H*. *avenae*, in the control of *H*. *avenae* through modifying the behavior of *H*. *avenae* around host roots.

## Supporting information

S1 Table Identification and quantitative analysis of low-molecular weight compounds in Bd21-3 root exudates. ^a^Numbers indicated the percentages of each compounds’ content in the two samples. ^b^Quantitative changes of each compounds after being heated were calculated using corresponding LM without heating as references. Values were means ± SE, n = 3.
(DOCX)

S2 Table Identification and quantitative analysis of low-molecular weight compounds in Zhengmai9023 root exudates. ^a^Numbers indicated the percentages of each compounds’ content in the two samples. ^b^Quantitative changes of each compounds after being heated were calculated using corresponding LM without heating as references. Values were means ± SE, n = 3.
(DOCX)

S3 Table Identification and quantitative analysis of low-molecular weight compounds in Heng4399 root exudates. ^a^Numbers indicated the percentages of each compounds’ content in the two samples. ^b^Quantitative changes of each compounds after being heated were calculated using corresponding LM without heating as references. Values were means ± SE, n = 3.
(DOCX)
