# Correction: Dihydroxyacetone of wheat root exudates serves as an attractant for *Heterodera avenae*

**DOI:** 10.1371/journal.pone.0251537

**Published:** 2021-05-06

**Authors:** Gaofeng Wang, Yunhe Wang, Hazem Abdelnabby, Xueqiong Xiao, Wenkun Huang, Deliang Peng, Yannong Xiao

An additional affiliation is missing for the third author. Hazem Abdelnabby is also affiliated with Department of Plant Protection, College of Agriculture, Benha University, Qaliubia, Egypt.

The following information is missing from the Funding statement: Yannong Xiao is supported by the National Key R & D Program of China (2018YFD0200500).

## References

[1] Wang G, Wang Y, Abdelnabby H, Xiao X, Huang W, Peng D, et al. (2020) Dihydroxyacetone of wheat root exudates serves as an attractant for *Heterodera avenae*. PLoS ONE 15(7): e0236317. 10.1371/journal.pone.0236317 32702002 PMC7377440

