# Supplementary material for: Dihydroxyacetone of wheat root exudates serves as an attractant for Heterodera avenae
Source: PLoS One. 2020 Jul 23;15(7):e0236317. doi: 10.1371/journal.pone.0236317 (PMC7377440; doi:10.1371/journal.pone.0236317)
Supplement: S1 Table — aNumbers indicated the percentages of each compounds’ content in the two samples. bQuantitative changes of each compounds after being heated were calculated using corresponding LM without heating as references. Values were means ± SE, n = 3. (DOCX) [file pone.0236317.s001.docx]

**S1 Table. Identification and quantitative analysis of LM in Bd21-3 root exudate**

| **Category** | **Compound** | **Molecular formula** | **Heat-treatment (%)^a^** | | **Quantitative change (%)^b^** |
| --- | --- | --- | --- | --- | --- |
|  |  |  | **LM without heating** | **LM heated at 100 ℃** |  |
| Organic acid | Rhein sulfate | C_15_H_8_O_9_S | 0.16 ± 0.04 | 0.04 ± 0.02 | 33.33 |
|  | 1-Aminocyclopropane-1-carboxylic acid | C_4_H_7_NO_2_ | 1.36 ± 0.37 | 0.00 ± 0.00 | -100.00 |
|  | Glyceric acid | C_3_H_6_O_4_ | 0.08 ± 0.04 | 0.00 ± 0.00 | -100.00 |
|  | DL-erythronic acid | C_4_H_8_O_5_ | 0.05 ± 0.02 | 0.08 ± 0.03 | 0.00 |
|  | Dimethylglycine | C_4_H_9_NO_2_ | 0.06 ± 0.02 | 0.06 ± 0.03 | 20.00 |
|  | Glutaconic acid | C_5_H_6_O_4_ | 0.05 ± 0.03 | 0.07 ± 0.03 | 16.67 |
|  | Terephthalic acid | C_8_H_6_O_4_ | 0.07 ± 0.02 | 0.05 ± 0.02 | 0.00 |
|  | Nalidixic acid | C_12_H_12_N_2_O_3_ | 0.07 ± 0.04 | 0.06 ± 0.02 | -14.29 |
|  | Fumaric acid | C_4_H_4_O_4_ | 0.06 ± 0.03 | 0.00 ± 0.00 | -100.00 |
|  | alpha-hydroxyisobutyrate | C_4_H_8_O_3_ | 0.48 ± 0.26 | 0.05 ± 0.02 | -16.67 |
|  | 2-methoxy-hexadecanoic acid | C_17_ H_34_ O_3_ | 0.06 ± 0.02 | 0.54 ± 0.26 | 12.50 |
|  | Parahydroxyphenylacetic acid | C_8_ H_8_ O_3_ | 6.70 ± 0.39 | 0.08 ± 0.04 | 33.33 |
|  | 3-Methyl-2-oxovaleric acid | C_6_ H_10_ O_3_ | 0.08 ± 0.03 | 4.94 ± 0.23 | -26.27 |
|  | Isovaleric acid | C_5_ H_10_ O_2_ | 0.11 ± 0.05 | 0.00 ± 0.00 | -100.00 |
|  | 9,12,14-octadecatrienoic acid | C_18_ H_30_ O_2_ | 0.04 ± 0.03 | 0.09 ± 0.03 | -18.18 |
|  | m-Hydroxyphenylpyruvic acid | C_9_ H_8_ O_4_ | 0.06 ± 0.04 | 0.00 ± 0.00 | -100.00 |
|  | Indole-3-carboxylic acid | C_9_ H_7_NO_2_ | 0.25 ± 0.12 | 0.07 ± 0.03 | 16.67 |
|  | Benzoic acid | C_8_ H_8_O_2_ | 0.04 ± 0.03 | 0.22 ± 0.14 | -12.00 |
|  | Phenylacetic acid | C_8_ H_8_ O_2_ | 0.05 ± 0.03 | 0.00 ± 0.00 | -100.00 |
|  | 2,5-decadienoic acid | C_10_ H_16_ O_2_ | 0.04 ± 0.01 | 0.04 ± 0.03 | -20.00 |
|  | 7-Hydroxyoctanoic acid | C_8_ H_16_ O_3_ | 0.04 ± 0.03 | 0.00 ± 0.00 | -100.00 |
|  | Dodecanedioic acid | C_12_ H_22_ O_4_ | 0.09 ± 0.04 | 0.05 ± 0.02 | 25.00 |
|  | 2-methyl-tridecanedioic acid | C_14_ H_26_ O_4_ | 0.07 ± 0.04 | 0.08 ± 0.02 | -11.11 |
|  | 3-methyl-heptanoic acid | C_8_ H_16_ O_2_ | 0.66 ± 0.02 | 0.42 ± 0.09 | 500.00 |
|  | Embelin | C_17_ H_26_ O_4_ | 0.07 ± 0.04 | 0.60 ± 0.01 | -9.09 |
|  | 4,12-dihydroxy-hexadecanoic acid | C_16_ H_32_ O_4_ | 0.71 ± 0.3 | 0.07 ± 0.03 | 0.00 |
|  | (+)-6-methyl caprylic acid | C_9_ H_18_ O_2_ | 13.04 ± 0.61 | 0.90 ± 0.43 | 26.76 |
|  | Methylprednisolone succinate | C_26_ H_34_ O_8_ | 0.21 ± 0.02 | 11.02 ± 0.48 | -15.49 |
|  | 3-methyl-nonanoic acid | C_10_ H_20_ O_2_ | 0.10 ± 0.05 | 0.32 ± 0.03 | 52.38 |
|  | 3R-hydroxy-tetradecanoic acid | C_14_ H_28_ O_3_ | 0.32 ± 0.13 | 0.12 ± 0.03 | 20.00 |
|  | 3-hydroxy-pentadecanoic acid | C_15_ H_30_ O_3_ | 0.17 ± 0.08 | 0.23 ± 0.08 | -28.13 |
|  | Apionic acid | C_5_ H_10_ O_6_ | 0.08 ± 0.05 | 0.11 ± 0.06 | -35.29 |
|  | 2,6-dimethyl-undecanoic acid | C_13_ H_26_ O_2_ | 0.30 ± 0.18 | 0.12 ± 0.03 | 50.00 |
|  | 3-hydroxy-hexadecanoic acid | C_16_ H_32_ O_3_ | 0.14 ± 0.12 | 0.32 ± 0.25 | 6.67 |
|  | 2-pentadecenoic acid | C_15_ H_28_ O_2_ | 0.09 ± 0.01 | 0.18 ± 0.06 | 28.57 |
|  | 4,8-dimethyl-dodecanoic acid | C_14_ H_28_ O_2_ | 0.05 ± 0.02 | 0.16 ± 0.02 | 77.78 |
| Aldehyde/Ketone/Phenol | 2-Keto-glutaramic acid | C_5_H_7_NO_4_ | 1.05 ± 0.11 | 0.00 ± 0.00 | -100.00 |
|  | Dihydroxyacetone | C_3_H_6_O_3_ | 0.10 ± 0.05 | 0.73 ± 0.02 | -30.48 |
|  | Phenol | C_6_ H_6_ O | 0.07 ± 0.04 | 0.00 ± 0.00 | -100.00 |
|  | 2,4-heptadienal | C_7_H_10_O | 0.25 ± 0.01 | 0.08 ± 0.03 | 14.29 |
|  | Diethylpropion | C_13_H_19_NO | 0.15 ± 0.07 | 0.22 ± 0.01 | -12.00 |
|  | Ethisterone | C_21_H_28_O_2_ | 0.12 ± 0.04 | 0.18 ± 0.06 | 20.00 |
|  | KOBUSONE | C_14_H_22_O_2_ | 0.03 ± 0.01 | 0.16 ± 0.04 | 33.33 |
|  | Mannitol | C_6_ H_14_O_6_ | 0.28 ± 0.13 | 0.00 ± 0.00 | -100.00 |
|  | Indole | C_8_H_7_N | 0.10 ± 0.04 | 0.03 ± 0.02 | -89.29 |
| Carbohydrate/Amino acid | D-Ribose | C_5_H_10_O_5_ | 0.04 ± 0.02 | 0.07 ± 0.02 | -30.00 |
|  | S-(Carboxymethyl)-L-cysteine | C_5_H_9_NO_4_S | 0.06 ± 0.03 | 0.00 ± 0.00 | -100.00 |
|  | N-Acetyl-L-glutamic acid | C_7_H_11_NO_5_ | 0.11 ± 0.04 | 0.00 ± 0.00 | -100.00 |
|  | Pyroglutamic acid | C_5_H_7_NO_3_ | 0.20 ± 0.08 | 0.14 ± 0.04 | 27.27 |
|  | Cystamine | C_4_ H_12_ N_2_ S_2_ | 0.73 ± 0.04 | 0.11 ± 0.05 | -45.00 |
| Others | Cyclophosphamide | C_7_H_15_C_l2_N_2_O_2_P | 0.17 ± 0.05 | 0.88 ± 0.03 | 20.55 |
|  | Phosphoric acid | H_3_O_4_P | 0.05 ± 0.02 | 0.22 ± 0.05 | 29.41 |
|  | Gibberellin A8 | C_19_ H_24_ O_7_ | 0.07 ± 0.03 | 0.00 ± 0.00 | -100.00 |
|  | HEMATEIN | C_16_H_12_O_6_ | 0.09 ± 0.05 | 0.00 ± 0.00 | -100.00 |
|  | Gluconolactone | C_6_H_10_O_6_ | 0.04 ± 0.02 | 0.00 ± 0.00 | -100.00 |
|  | 4-Hydroxypyridine | C_5_H_5_NO | 0.02 ± 0.01 | 0.05 ± 0.02 | 25.00 |
|  | Pyridine | C_5_H_5_N | 0.16 ± 0.07 | 0.00 ± 0.00 | -100.00 |
|  | Thymine | C_5_ H_6_ N_2_ O_2_ | 0.42 ± 0.02 | 0.20 ± 0.06 | 25.00 |
|  | Sulfaphenazole | C_15_H_14_N_4_O_2_S | 0.05 ± 0.04 | 0.54 ± 0.02 | 28.57 |
|  | Benzyl alcohol | C_7_ H_8_ O | 0.16 ± 0.04 | 0.06 ± 0.02 | 20.00 |

Note: ^a^Numbers indicated the percentages of each compounds' content in the two samples. ^b^Quantitative changes of each compounds after being heated were calculated using corresponding LM without heating as references. Values were means ± SE, n = 3.
