# Supplementary material for: Dihydroxyacetone of wheat root exudates serves as an attractant for Heterodera avenae
Source: PLoS One. 2020 Jul 23;15(7):e0236317. doi: 10.1371/journal.pone.0236317 (PMC7377440; doi:10.1371/journal.pone.0236317)
Supplement: S2 Table — aNumbers indicated the percentages of each compounds’ content in the two samples. bQuantitative changes of each compounds after being heated were calculated using corresponding LM without heating as references. Values were means ± SE, n = 3. (DOCX) [file pone.0236317.s002.docx]

**S2 Table. Identification and quantitative analysis of LM in Zhengmai9023 root exudate**

| **Category** | **Compound** | **Molecular formula** | **Heat-treatment (%)^a^** | | **Quantitative change (%)^b^** |
| --- | --- | --- | --- | --- | --- |
|  |  |  | **LM without heating** | **LM heated at 100 ℃** |  |
| Organic acid | Lauric acid | C_12_ H_24_ O_2_ | 1.89 ± 0.87 | 1.09 ± 0.48 | -42.33 |
|  | 3,12-dihydroxypalmitic acid | C_16_ H_32_ O_4_ | 0.28 ± 0.11 | 0.31 ± 0.06 | 10.71 |
|  | Stearic acid | C_18_ H_36_ O_2_ | 12.99 ± 3.19 | 3.66 ± 0.85 | -71.82 |
|  | cyclandelate | C_17_ H_24_ O_3_ | 0.12 ± 0.05 | 0.09 ± 0.04 | -25.00 |
|  | Apionic acid | C_5_ H_10_ O_6_ | 0.29 ± 0.06 | 0.16 ± 0.05 | -44.83 |
|  | Reserpic acid | C_22_ H_28_ N_2_ O_5_ | 0.13 ± 0.05 | 0.12 ± 0.06 | -7.69 |
|  | Methylprednisolone succinate | C_26_ H_34_ O_8_ | 1.75 ± 0.48 | 0.12 ± 0.06 | -93.14 |
|  | Embelin | C_17_ H_26_ O_4_ | 0.67 ± 0.06 | 0.39 ± 0.05 | -41.79 |
|  | 9,12,14-octadecatrienoic acid | C_18_ H_30_ O_2_ | 0.36 ± 0.07 | 0.17 ± 0.05 | -52.78 |
|  | (+)-6-methyl caprylic acid | C_9_ H_18_ O_2_ | 2.11 ± 0.94 | 1.10 ± 0.24 | -47.87 |
|  | 5-methyl-2E-tridecenoic acid | C_14_ H_26_ O_2_ | 0.96 ± 0.09 | 0.50 ± 0.09 | -47.92 |
|  | 4,8-dimethyl-dodecanoic acid | C_14_ H_28_ O_2_ | 2.82 ± 0.37 | 1.23 ± 0.27 | -56.38 |
|  | 3-methyl-nonanoic acid | C_10_ H_20_ O_2_ | 0.99 ± 0.50 | 0.71 ± 0.33 | -28.28 |
|  | 3-methyl-heptanoic acid | C_8_ H_16_ O_2_ | 1.00 ± 0.15 | 0.46 ± 0.11 | -54.00 |
|  | 3R-hydroxy-tetradecanoic acid | C_14_ H_28_ O3 | 0.16 ± 0.03 | 0.12 ± 0.03 | -25.00 |
|  | 2-pentadecenoic acid | C_15_ H_28_ O_2_ | 0.42 ± 0.15 | 0.23 ± 0.11 | -45.24 |
|  | 1,11-Undecanedicarboxylic acid | C_13_ H_24_ O_4_ | 0.14 ± 0.08 | 0.00 ± 0.00 | -100.00 |
|  | Panaxytriol | C_17_ H_26_ O_3_ | 2.13 ± 0.56 | 2.20 ± 0.51 | 3.29 |
| Aldehyde/Ketone/Phenol | Diethylpropion | C_13_ H_19_ N O | 0.37 ± 0.04 | 0.20 ± 0.03 | -45.95 |
|  | Dihydroxyacetone | C_3_ H_6_ O_3_ | 0.57 ± 0.20 | 0.00 ± 0.00 | -100.00 |
|  | Levonorgestrel acetate | C_23_ H_30_ O_3_ | 1.21 ± 0.40 | 0.76 ± 0.14 | -37.19 |
|  | Ethisterone | C_21_ H_28_ O_2_ | 0.36 ± 0.08 | 0.20 ± 0.10 | -44.44 |
|  | KOBUSONE | C_14_ H_22_ O_2_ | 0.35 ± 0.05 | 0.22 ± 0.07 | -37.14 |
| Other | Cyclophosphamide | C_7_H_15_C_l2_N_2_O_2_P | 0.48 ± 0.12 | 0.00 ± 0.00 | -100.00 |
|  | METAXALONE | C_12_ H_15_ N O_3_ | 0.18 ± 0.03 | 0.67 ± 0.2 | 272.22 |

Note: ^a^Numbers indicated the percentages of each compounds' content in the two samples. ^b^Quantitative changes of each compounds after being heated were calculated using corresponding LM without heating as references. Values were means ± SE, n = 3.
