# Supplementary material for: Dihydroxyacetone of wheat root exudates serves as an attractant for Heterodera avenae
Source: PLoS One. 2020 Jul 23;15(7):e0236317. doi: 10.1371/journal.pone.0236317 (PMC7377440; doi:10.1371/journal.pone.0236317)
Supplement: S3 Table — aNumbers indicated the percentages of each compounds’ content in the two samples. bQuantitative changes of each compounds after being heated were calculated using corresponding LM without heating as references. Values were means ± SE, n = 3. (DOCX) [file pone.0236317.s003.docx]

**S3 Table. Identification and quantitative analysis of LM in Heng4399 root exudate**

| **Category** | **Compound** | **Molecular formula** | **Heat-treatment (%)^a^** | | **Quantitative change (%)^b^** |
| --- | --- | --- | --- | --- | --- |
|  |  |  | **LM without heating** | **LM heated at 100 ℃** |  |
| Organic acid | Quinic acid | C_7_ H_12_O_6_ | 0.10 ± 0.04 | 0.08 ± 0.01 | -20.00 |
|  | 1,11-Undecanedicarboxylic acid | C_13_ H_24_O_4_ | 0.10 ± 0.03 | 0.13 ± 0.03 | 30.00 |
|  | 3-methyl-heptanoic acid | C_8_ H_16_O_2_ | 0.67 ± 0.17 | 0.80 ± 0.08 | 19.40 |
|  | Embelin | C_17_ H_26_O_4_ | 1.00 ± 0.08 | 0.74 ± 0.04 | -26.00 |
|  | (+)-6-methyl caprylic acid | C_9_ H_18_O_2_ | 1.51 ± 0.33 | 1.63 ± 0.40 | 7.95 |
|  | D-Phenyllactic acid | C_9_ H_10_O_3_ | 0.13 ± 0.07 | 0.00 ± 0.00 | -100.00 |
|  | 3,12-dihydroxy palmitic acid | C_16_ H3_2_O_4_ | 0.16 ± 0.04 | 0.18 ± 0.04 | 12.50 |
|  | Reserpic acid | C_22_ H_28_N_2_O_5_ | 0.18 ± 0.05 | 0.12 ± 0.05 | -33.33 |
|  | 3R-hydroxy-tetradecanoic acid | C_14_ H_28_ O_3_ | 0.11 ± 0.03 | 0.12 ± 0.05 | 9.09 |
|  | Methylprednisolone succinate | C_26_ H_34_O_8_ | 3.18 ± 0.22 | 2.12 ± 0.21 | -33.33 |
|  | 3-methyl-nonanoic acid | C_10_ H_20_ O_2_ | 0.59 ± 0.16 | 0.71 ± 0.19 | 20.34 |
|  | 2-pentadecenoic acid | C_15_ H_28_ O_2_ | 0.26 ± 0.07 | 0.33 ± 0.06 | 26.92 |
|  | 9,12,14-octadecatrienoic acid | C_18_ H_30_ O_2_ | 0.23 ± 0.06 | 0.29 ± 0.09 | 26.09 |
|  | 4,8-dimethyl-dodecanoic acid | C14 H28 O2 | 2.09 ± 0.02 | 2.18 ± 0.02 | 4.31 |
|  | 2,6-dimethyl-undecanoic acid | C_13_ H_26_O_2_ | 0.18 ± 0.06 | 0.00 ± 0.00 | -100.00 |
|  | Lauric acid | C_12_ H_24_ O_2_ | 1.45 ± 0.30 | 1.48 ± 0.35 | 2.07 |
|  | Apionic acid | C_5_ H_10_ O_6_ | 0.23 ± 0.05 | 0.23 ± 0.03 | 0.00 |
| Aldehyde/Ketone/Phenol | Ethisterone | C_21_ H_28_ O_2_ | 0.30 ± 0.07 | 0.26 ± 0.10 | -13.33 |
|  | Levonorgestrel acetate | C_23_ H_30_ O_3_ | 0.74 ± 0.41 | 0.82 ± 0.47 | 10.81 |
|  | Dihydroxyacetone | C_3_ H_6_O_3_ | 0.44 ± 0.08 | 0.00 ± 0.00 | -100.00 |
|  | Pyrocatechol | C_6_ H_6_ O_2_ | 0.10 ± 0.03 | 0.11 ± 0.03 | 10.00 |
|  | 15-HydroxyCyproterone | C_22_ H_27_ClO_4_ | 0.20 ± 0.03 | 0.24 ± 0.08 | 20.00 |
|  | 9-ketopalmitic acid | C_16_ H_30_O_3_ | 0.10 ± 0.04 | 0.17 ± 0.03 | 70.00 |
|  | KOBUSONE | C_14_ H_22_O_2_ | 0.27 ± 0.06 | 0.28 ± 0.10 | 3.70 |
|  | Diethylpropion | C_13_H_19_ NO | 0.41 ± 0.03 | 0.32 ± 0.02 | -21.95 |
| Other | Tyrosine | C_9_ H_11_ N O_3_ | 0.19 ± 0.05 | 0.00 ± 0.00 | -100.00 |
|  | Sulfaphenazole | C_15_ H_14_N_4_O_2_S | 0.84 ± 0.14 | 1.01 ± 0.25 | 20.24 |
|  | Metanephrine | C_10_ H_15_NO_3_ | 0.20 ± 0.05 | 0.12 ± 0.04 | -40.00 |
|  | Phosphoric acid | H_3_ O_4_ P | 2.35 ± 0.36 | 2.53 ± 0.61 | 7.66 |

Note: ^a^Numbers indicated the percentages of each compounds' content in the two samples. ^b^Quantitative changes of each compounds after being heated were calculated using corresponding LM without heating as references. Values were means ± SE, n = 3.
